# Outcome and quality of life after intracranial meningioma surgery in cats

**DOI:** 10.1177/1098612X231194425

**Published:** 2023-10-23

**Authors:** Lydia Koch, Alexander Tichy, Gabriele Gradner

**Affiliations:** 1 Department for Small Animals and Horses, Small Animal Surgery, University of Veterinary Medicine Vienna, Vienna, Austria; 2 Bioinformatics and Biostatistics Platform, Department of Biomedical Sciences, University of Veterinary Medicine Vienna, Vienna, Austria

**Keywords:** Meningioma, intracranial surgery, neurosurgery, quality of life

## Abstract

**Objectives:**

The present study aimed to evaluate the postoperative quality of life (QOL) after surgery for the treatment of intracranial meningioma in cats

**Methods:**

The study included 14 cases that underwent craniotomy from May 2009 to March 2021. Owners were contacted via telephone after a median time of 967 (range 227–4209) days after surgery and surveyed with a specially designed questionnaire that consisted of three domains, subdivided into different items. Physical behaviour, including general condition, food intake, mobility and overall impression, was evaluated from 0, reflecting the worst status, to 10, reflecting the best. The development of preoperative existing clinical signs, seizures and concurrent medication were evaluated individually for each patient. The time span necessary for the improvement of each item was recorded. Finally, satisfaction about the decision for surgery was ranked from 0 to 10.

**Results:**

Thirteen questionnaires were completed. Three cats were evaluated twice owing to revision surgery. Owners reported a statistically significant (*P* <0.001) improvement from immediately after the operation to 240 days after surgery. Preoperative clinical signs resolved in 95% of cases. All questioned owners would opt for surgery again.

**Conclusions and relevance:**

QOL after surgery for intracranial meningioma in cats seems encouraging regarding our study. Nevertheless, limitations, such as small sample size, recall bias, lack of a control group and validation of the questionnaire, need to be kept in mind when interpreting the results.

## Introduction

Treatment possibilities for several diseases in veterinary medicine have increased, enabling a markedly prolonged survival time.^1,2^ This might be associated with an increase in possible morbidities impacting quality of life (QOL) and the need for balancing these with QOL.^1,2^ QOL assessment has gained importance for decision-making and assessment of whether a dog’s or cat’s life is still worth living.^1,3–10^ Depending on the disease, different questionnaires have been developed in human medicine and are constantly adapted.^1,3–13^ Some of them are readily used, based on direct dialogues between surgeons and patients. They are useful regarding long-term outcomes and can assist in decision-making.^2^ This is important especially for patients receiving palliative treatment, where the goal is retaining or improving their QOL instead of excessively elongating their lifespan.^2^

Because animals are incapable of providing subjective experiences by themselves, no consensus has been reached regarding questions about how to assess their QOL.^1–3,5,7,9,10,13^ Focusing on aspects of life that are important for the animal and evaluating patient-related outcomes beyond clinical parameters have been suggested.^2^

In veterinary medicine, studies evaluating QOL of dogs and cats have been performed for cardiac disease, chronic degenerative joint disease, pain secondary to cancer and injuries of the spinal cord.^3–5,9,10,13^ Besides disease-related questions, the most common questions are about food intake, behaviour and mobility.^3,4,9,10^

Assessments of long-term QOL after meningioma surgery in cats or dogs have, to our knowledge, not yet been performed.

Because the reported age for cats presented with intracranial meningioma is generally older than 10 years, there might be a discussion about the usefulness of, and the QOL after, surgery.^14,15^ The most common clinical signs in these cats are altered consciousness, seizures, circling, ataxia, decreased vision to blindness, and unspecific signs such as lethargy and anorexia in approximately 21% of feline patients.^14–18^ Usually, they are progressive with severity depending on location, growth rate, size, amount of peritumoral oedema and intracranial pressure.^16,19,20^

The present study aimed to evaluate the long-term postoperative outcome and QOL in cats after receiving surgery for the treatment of intracranial meningioma and to assist decision-making regarding whether to perform surgical treatment.

The questionnaire for the study was mostly based on that from Weiske et al.,^8^ which was developed to evaluate QOL in dogs with different types of intracranial disease.

Our hypothesis was that the cats would have a good long-term QOL and show improvement in preoperative clinical signs and aspects affecting their daily life after surgery.

## Materials and methods

### Patient selection

The patient database of the University of Veterinary Medicine in Vienna was searched for cats with histopathologically confirmed intracranial meningioma treated with craniotomy from May 2009 to March 2021. To meet the inclusion criteria, patients needed to have magnetic resonance imaging (MRI) performed prior to surgery.

### Data collection

For each included patient, their age, cause of presentation, preoperative neurological status, MRI findings, disease-related medications or therapies, and survival time were obtained.

To evaluate postoperative development, a standardised questionnaire, adapted from the study by Weiske et al,^8^ was developed. The number of questions asked was reduced, and questions were designed with respect to the most common preoperative changes noticed in all patients, as well as patient-specific clinical signs.

Owners of all cats receiving surgery for meningioma in the forementioned period of time and meeting the inclusion criteria were contacted via telephone in November 2020 to March 2021, and the time from surgery to survey was calculated. Questioning was performed to obtain information regarding the status of the first few days after discharge and the current status or the best status before death within one conversation. In the case of death, the cause and date were noted. Our questionnaire consisted of three domains, each subdivided into different items. For the first domain, physical behaviour, including general condition, food intake and mobility, was evaluated. General condition was ranked from lethargy to the cat’s normal behaviour. Food intake was screened from insufficient to sufficient. For mobility, the scale ranged from an inability to walk to a normal gait with the ability to jump. Grading was possible on a range from 0, reflecting the worst condition, to 10, reflecting the best.

The second domain evaluated disease-related changes and included the development of preoperative existing clinical signs, seizures and related medication. Questions regarding the improvement of preoperatively existing clinical signs were designed as closed ones and were personalised for each cat. The occurrence of postoperative seizures was evaluated for the period immediately after surgery and the current situation or situation before death. The necessity of medication for seizure control and the type of medication were obtained.

In the last domain, the overall impression was evaluated by asking whether the cat had more bad days than good days, or vice versa. Again, grading was possible within a range from 0 to 10.

Time until improvement in days was evaluated for every item.

Finally, owners were asked, without considering financial aspects, if they would choose to have surgery for their cat again, and 0 represented a strong ‘no’, while 10 represented a strong ‘yes’.

### Statistical analysis

Statistical analysis was performed using SPSS, version 19 (IBM Corp.). To evaluate the differences between values immediately after surgery and at the time of questioning, or before death, the Wilcoxon test was used. *P* <0.05 was considered statistically significant. For non-descriptive statistical analysis, only results from the first surgery were used, because taking a repetitive assessment for one cat into account would bear the risk of not being independent from the first one.

## Results

### Medical history

Fourteen cats, all domestic shorthair, with a median (range) age of 11 (5–14) years at the time of surgery, were included in the study. The gender distribution was equal, with seven males and seven females. The most common reason for initial patient presentation was a change of behaviour, in 11 cases (79%), followed by lethargy, in eight (57%) cases. Circling was reported in 5/14 (36%) cases, followed by seizures and undefined pain, each present in four cats (29%). Further clinical signs were disorientation, problems with coordination, anorexia and blindness in three cats (21%). Weakness, weight loss and anosmia were present in only two patients (14%) and the least common clinical signs were head tilt and ataxia, each in only one cat (7%).

### Neurological examination

Complete preoperative neurological examination was possible in 13/14 cases, because one had been sedated until surgery owing to epileptic seizures. For the remaining cats, the most common clinical finding was lethargy, in six (46%) patients, followed by hopping deficiencies, in five (38%) patients. Ataxia and reduced pupillary reflex were present in four (31%) cats. Three cats (23%) showed proprioceptive deficiencies, blindness and pacing. Weakness, an exaggerated patellar reflex, a reduced withdrawal reflex, a loss of face sensibility, head tilt and circling were each clinically present in two (15%) patients. The least common clinical findings were the inability to walk, an exaggerated extensor carpi radialis reflex, an exaggerated tibialis cranialis reflex, a reduced palpebral reflex and head turn, each present in one (8%) cat. The results of the neurological examination are listed in Table 1.

**Table 1:** Findings according to the preoperative neurological examination

Frequency of occurrence	Clinical signs
46%	Lethargy
38%	Hopping deficiencies
31%	Ataxia Reduced pupillary reflex
23%	Proprioceptive deficiencies Blindness Pacing
15%	Weakness Exaggerated patellar reflex Reduced withdrawal reflex Loss of face sensibility Head tilt Circling
8%	Inability to walk Exaggerated extensor carpi radialis reflex Exaggerated tibialis cranialis reflex Reduced palpebral reflex Head turn

### Tumour location

The most commonly affected region was the parietal lobe, in eight (57%) patients. The frontal lobe was affected in five (36%) cats, and the temporal lobe was affected in four (29%). In two (14%) patients, the occipital lobe was involved, the falx cerebri was involved in one (7%), and the tentorium cerebelli was involved in another (7%). In 6/14 cases (42%), the meningioma overlapped in three regions. One (7%) cat was diagnosed with multiple meningiomas: one in the parietal lobe and one in the temporal lobe.

### Surgery and postoperative treatment

Depending on the location of the tumour, surgery was performed either by a rostrotentorial or caudotentorial approach. It was performed by a European College of Veterinary Surgeons board-certified small-animal surgeon in all cases.

Postoperatively, all patients were transferred to an intensive care unit. Three out of 14 (21%) cases died within 4 days of surgery and were excluded from further outcome evaluation. One died 24 h after surgery because this cat required ventilation owing to haemoglobin desaturation and was subsequently euthanased at the owner’s request. Another cat died within 48 h of surgery after cardiopulmonary arrest without the recurrence of spontaneous circulation. The third cat was euthanased on day 4 after surgery owing to lung oedema and acute renal failure.

All remaining 11 cats were discharged. Gabapentin was continued after discharge in all remaining 11 (100%) patients. Nine cats (82%) received further treatment with prednisolone and an additional gastroprotective. Anticonvulsive treatment with phenobarbital and levetiracetam was continued in 2/11 (18%) discharged patients. One cat (9%) needed further treatment with tramadol because of pain.

### Pathohistological examination

The most common tumour type was transitional meningioma, in 6/14 (43%) cases. The second most common was the fibrous type, in 5/14 (36%) cases, followed by psammomatous (2/4; 14%) and meningothelial types (1/14; 7%).

### Outcome

The median (range) survival time was 861 (15–2064) days. Six out of the 11 cats (55%) were still alive at the time of survey. Of the five deceased cases, two were euthanased owing to multiple seizures (one surviving 1377 days and the other 15 days after the operation). One cat was euthanased 2064 days after surgery owing to multimorbidity, apathy and anorexia. Another cat developed transitional cell carcinoma of the urinary bladder and was euthanased 1215 days after meningioma surgery owing to problems with defecation, pollakiuria, pain and vomiting. Cause of death of the remaining cat could not be evaluated as the owner only responded with the year of death and did not answer further questions. This cat survived 474 days after surgery.

Three cats (27%) had recurrence at a median (range) of 851 (133–1778) days after their first surgery and received revision surgery. One of those cats received additional radiotherapy. All of those cats were still alive at the time of questioning.

### Questionnaire results

#### Descriptive analysis

The owners of 11 cats discharged from hospital were surveyed within a median (range) time from surgery to telephone survey of 967 (227–4209) days after the first surgery. The owners of three cats that received revision surgery owing to tumour regrowth were asked to answer for both surgeries separately, leading to a total of 14 questionnaires. Of those, one owner reported the year of death, but was not able to answer further questions. A full survey was therefore completed in 13/14 cases (93%).

Postoperative behaviour was rated with a mean (SD) of 6.5/10 (±2.9), which improved to 9.4/10 (±1.1) at the stage of full recovery. The mean (range) time to full recovery was 17 (5–60) days. Postoperative food intake was reported with a mean (SD) of 5.8/10 (±4.5) and improved to 10/10 (±0) after a mean (range) of 26 (2–60) days. Three owners needed to feed their cats with an oesophageal tube for a mean (range) time of 7 (2–28) days. The cats’ mobility after surgery was graded with an average of 6.1/10 and improved after a mean (range) of 73 (0–240) days to a mean (SD) of 8.7 (±2.0).

Overall postoperative impression was ranked with a mean (SD) of 7.3/10 (±2.6) and improved to 10/10 (±0) (*P* = 0.007) at the time of full recovery.

Preoperative existing clinical signs resolved in 95% of cases, with a calculated improvement of 100% for all clinical signs. In two patients, clinical signs improved markedly, but ataxia slightly persisted in both. One cat was deaf in one ear after surgery, according to the owner.

Two cases (14%) suffered from postoperative epileptic seizures. One of them was referred to an external veterinary hospital 15 days after surgery and was euthanased. In one cat, the seizures were associated with tumour regrowth 133 days after the first surgery and resolved after revision surgery. Two cats had no clinical signs when recurrence was observed through MRI, but developed seizures after the second surgery and were still receiving medication at the time of questioning.

Decision-making regarding the subsequent recovery and life quality of their cat was ranked out of 10 by all owners, including those of cats that had undergone revision surgery. The results are shown in Table 2.

**Table 2:** Evaluated items and results of descriptive analysis from the questionnaire

First domain			
Item	Score 1^*^	Score 2^†^	
Behaviour	6.5 (±2.9)	9.4 (±1,1)	
Food intake	5.8 (±4.5)	10 (±0)	
Mobility	6.1 (±3.0)	8.7 (±2.0)	
Second domain			
Preoperative clinical sign	Total	Improved	Percent
Behaviour change	8	8	100
Lethargy	7	7	
Circling	4	4	
Seizures	3	3	
Undefined pain	3	3	
Disorientation	2	2	
Coordination problems	3	3	
Anorexia	3	3	
Blindness	2	2	
Weakness	2	2	
Weight loss	2	2	
Anosmia	2	2	
Head tilt	1	1	
Ataxia	1	1	
	Total	Resolved	Percent
Behaviour change	8	8	95
Lethargy	7	7	
Circling	4	4	
Seizures	3	3	
Undefined pain	3	3	
Disorientation	2	2	
Coordination problems	3	2	
Anorexia	3	3	
Blindness	2	2	
Weakness	2	2	
Weight loss	2	2	
Anosmia	2	2	
Head tilt	1	1	
Ataxia	1	0	
Seizures	Yes	No	Percent
Postoperative	2	12	14
Third domain			
	Score 1^*^	Score 2^†^	
Overall impression	7.3 (±2.6)	10 (±0)	
Decision-making owner			
	Score	Answers	Percent
	0–9	0/14	0
	10	14/14	100

Scores for pre- and postoperative evaluations are listed for the first and third domains. Values in parentheses represent the SD. Percentages are listed for the improvement or resolution of preoperative clinical signs and seizures

* Score 1: average preoperative score

† Score 2: average postoperative score

#### Pre- and postoperative comparisons

Results from 10 questionnaires evaluating cats after receiving one surgery were eligible for comparison with the Wilcoxon test. All evaluated items showed a statistically significant difference between postoperative state and state at full recovery. The *P* value for the difference in behaviour postoperatively and at the state of full recovery was 0.011. For food intake and mobility, *P* values were 0.042 and 0.043, respectively. For overall impression, the *P* value was 0.027.

## Discussion

Our hypothesis was that cats undergoing craniotomy for treatment of intracranial meningioma would have a high, long-term QOL and show improvement in preoperative clinical signs and aspects affecting their daily lives after surgery.

All cats improved in terms of behaviour, food intake, mobility and overall impression after surgery. In all patients, preoperative existing clinical signs resolved or at least markedly improved. Where causes for postoperative seizures could be obtained, they were either associated with tumour regrowth or revision surgery.

Accordingly, we accept our study hypothesis.

All questioned owners reported that they would opt for surgery again for treatment of intracranial meningioma in their cats.

A median age of 11.6 years at time of surgery in the present study corresponds to findings in previous work.^14,15^

The most common clinical signs leading to initial patient presentation were behaviour change, apathy, seizures and circling, which have also been reported as the most common findings in studies by Troxel et al^14^ and Nafe.^21^ No study could be found reporting undefined pain as a clinical sign of intracranial meningioma, which was present in 31% of cases in our study. It remains unclear whether this clinical sign had been classified as behaviour change in previous work or indeed has not yet been reported. Nevertheless, in cats presenting with pain of unknown origin, intracranial changes should be considered a possible cause.

Regarding the MRI findings, regions most commonly affected by meningioma were the parietal, frontal and temporal lobes in that order, which is comparable to those reported in previously published studies.^14,17,18,21–24^

The median survival time of 861 days in the present study is also comparable to previous studies, which reported 665 and 685 days.^14,17^

A drawback of the present study is the small sample size, which needs to be considered when interpreting the results. A prospective design with given intervals of clinical neurological and MRI re-examination would lead to more data. The importance of the latter is reflected by the possibility of tumour regrowth without neurological deficiencies or clinical changes, which was present in two cats in our study and has also been reported in a study by Forterre et al.^17^ It would be useful to perform MRI examinations regularly after surgery to detect regrowth as soon as possible and thus perform revision surgery, as in human medicine, where follow-up MRI examinations are performed every 3–6 months.^25^

The evaluation of postoperative development and QOL over the long term solely by the owner is a further limitation. An evaluation of progress after surgery by veterinary professionals at given intervals would have led to additional objective information. Nevertheless, relying solely on clinical assessments and biological parameters (eg, blood work) of an animal is not sufficiently accurate to fully assess its QOL because this does not take important parts of the animal’s life into account.^2^ Information provided by the owner is indispensable because they will have more experience with the individuals' needs and habits.^2,26^

A major limitation of the present study is the time frame between surgery and survey, which ranged up to 4209 days and was not equal for all cases. Furthermore, owners were asked to recall their cat’s status after being discharged and, if the cat had already died, before death, which, owing to the given time frame, could have led to a considerable recall bias. This highlights the importance for future studies to be designed in a prospective way, with prefixed intervals for owners to be contacted to more reliably evaluate the animal’s development.

Scales for the evaluation of QOL are highly variable, ranging from 0 to 3 or from 0 to 100.^1,4,7–9,27^ We used a scale ranging from 0 to 10, as suggested in a study by Lynch et al.^1^

A very important part of QOL evaluation in humans is an evaluation of mental health status and emotional function.^11,27–32^ This cannot be assessed in a comparable way in animals and therefore relies on interpretations of external parameters by owners or veterinarians.^1,2^ Parameters may include a willingness to go for a walk, to play and to interact with the owner and other animals, but these parameters, by the nature of the questions, have mostly been used to evaluate dogs’ QOL.^1,5,8–10,13^ Therefore, more precise and cat-specific questions should be established in further studies to gain more information. To evaluate to what extent a domain measures what it is intended to measure, questionnaires can be validated.^33^ Usually, a group of animals with a disease to be evaluated, and also control groups, are assessed with the same questionnaire.^9,10,33^ Because the present study was designed to solely evaluate cats that have undergone surgery for meningioma and there was a lack of a control group, this has not been carried out. It represents a further study limitation and should be performed in future studies.

Nevertheless, the philosophical question of how accurate proxy reporting is remains unsolved.

Food intake and mobility can be more accurately answered by owners, but neurological deficiencies such as mild ataxia or proprioceptive deficits might not be observed. Therefore, a neurological evaluation at given intervals by a veterinarian would add additional information. This also applies to questions regarding preoperative clinical signs.

Obvious clinical signs such as complete blindness or circling may be accurately evaluated by the owners, but the slight persistence of, for example, vestibular deficits might only be detected by specialists.

Even if 100% of the asked owners would choose surgery for treatment of intracranial meningioma again, it has to be kept in mind that three cats that died while still inpatients after surgery have been excluded from further evaluation. Therefore, there is no information about whether these owners would also opt for surgery again. It also needs to be kept in mind that owners choosing surgery as therapy might be more motivated and positive, possibly reflecting the positive answers regarding QOL of their cats after surgery. To compare outcomes between surgically treated patients and patients not undergoing treatment or other treatment modalities, such as radiation only, comparison with a control group would have been beneficial.

## Conclusions

Allowing for study limitations, the findings regarding both development and QOL after surgery for intracranial meningioma appear to be encouraging, and surgery should be considered in these cases.
